# Organizational characteristics of HIV/syphilis testing services for men who have sex with men in South China: a social entrepreneurship analysis and implications for creating sustainable service models

**DOI:** 10.1186/s12879-014-0601-5

**Published:** 2014-11-25

**Authors:** Joseph D Tucker, Kathryn E Muessig, Rosa Cui, Cedric H Bien, Elaine J Lo, Ramon Lee, Kaidi Wang, Larry Han, Feng-Ying Liu, Li-Gang Yang, Bin Yang, Heidi Larson, Rosanna W Peeling

**Affiliations:** University of North Carolina Project-China, Number 2 Lujing Road, Guangzhou, 510095 China; University of North Carolina Gillings School of Global Public Health, 135 Dauer Drive, Chapel Hill, 27599 NC USA; Columbia College of Physicians and Surgeons, 630 West 168th Street, New York, 10032 NY USA; Icahn School of Medicine at Mt. Sinai, 1428 Madison Ave, New York, 10029 USA; Harvard Medical School, 25 Shattuck Street, Boston, 02115 MA USA; Stanford University School of Medicine, 450 Serra Mall, Stanford, 94305 CA USA; Guangdong Provincial Centers for Skin Diseases and STI Control, Number 2 Lujing Road, Guangzhou, 510095 China; London School of Hygiene and Tropical Medicine, Keppel Street, London, WC1E 7HT UK

**Keywords:** China, HIV, Syphilis, MSM, Social entrepreneurship, Social enterprise

## Abstract

**Background:**

UNAIDS has called for greater HIV/syphilis testing worldwide just as local HIV/syphilis testing programs are cut or altered. New models are needed to make HIV/syphilis testing services sustainable while retaining their essential public health function. Social entrepreneurship, using business principles to promote a social cause, provides a framework to pilot programs that sustainably expand testing. Drawing on fieldwork in two South Chinese cities, we examined organizational and financial characteristics of current HIV/syphilis testing systems for men who have sex with men (MSM) in addition to new pilot programs focused on revenue-generation for sustainability.

**Methods:**

We undertook a qualitative study to explore organizational and financial characteristics of HIV/syphilis testing for MSM. Data were collected from men who have sex with men and policy stakeholders in Guangzhou and Hong Kong. Framework analysis was used to identify themes and then code the data.

**Results:**

Our qualitative research study included MSM and policy stakeholders (n = 84). HIV/syphilis testing services were implemented at a wide range of organizations which we grouped broadly as independent community-based organizations (CBOs), independent clinics, and hybrid CBO-clinic sites. From an organizational perspective, hybrid CBO-clinic sites offered the inclusive environment of an MSM CBO linked to the technical capacity and trained staff of a clinic. From a financial perspective, stakeholders expressed concern about the sustainability and effectiveness of sexual health services reliant on external funding. We identified four hybrid CBO-clinic organizations that launched pilot testing programs in order to generate revenue while expanding HIV testing.

**Conclusion:**

Many MSM CBOs are searching for new organizational models to account for decreased external support. Hybrid CBO-clinic organizations create a strong foundation to increase HIV/syphilis testing using social entrepreneurship models in China.

**Electronic supplementary material:**

The online version of this article (doi:10.1186/s12879-014-0601-5) contains supplementary material, which is available to authorized users.

## Background

Unsafe sex is the second most common cause of death and disability in the world [1], resulting in an estimated 340 million preventable sexually transmitted diseases (STDs) each year [2]. Biological and social forces converge so that key populations such as men who have sex with men (MSM) and sex workers acquire a disproportionate burden of STDs [3], especially syphilis. Marshaling public resources for what are often private HIV/syphilis services among stigmatized key populations has been historically challenging [4], exacerbated now in the context of global financial uncertainty. The Global Fund to Fight AIDS, Tuberculosis, and Malaria is constricting and other international donors are reducing or eliminating HIV/STD programs in low and middle income nations [5]. As a result, over two-thirds of MSM globally are not reached by HIV prevention services [6]. Test-associated stigma [7] and mistrust of public sector HIV testing services [8] persist. In China, despite strong government resources to promote HIV testing, direct support from government public health agencies to community-based organizations (CBOs) has been challenging [9],[10]. In order to meet the UNAIDS target for 15 million HIV-infected individuals on antiretroviral therapy by 2015 [11], sustainable HIV/syphilis service models are needed. Sustainable models are critical for ensuring access across the cascade of HIV/syphilis services [12], establishing trusting long-term relationships between physicians and patients [13], and ultimately providing cost-effective care [14].

Social entrepreneurship is a potentially powerful framework that could be used to achieve sustainable HIV/syphilis services. Broadly speaking, social entrepreneurship is the use of business principles to advance a social cause, ranging from micro-enterprise efforts to small business development [15]. Social entrepreneurship for sexual health (SESH) model programs generate revenues that are re-invested in HIV/syphilis programs. A set of core values defines SESH programs and can be used to distinguish these programs from conventional approaches (Table 1). Such SESH models could make community-based organization (CBO) services less reliant on external resources and more responsive to the communities they serve [16]. For example, Mechai Viravaidya's CBO, Population Development Association, raises money through revenue-maximizing businesses (e.g., restaurant and resort) which is then re-invested in family planning services and condom promotion, a model that has been recognized as a UNAIDS best practice [17]. Another example of SESH is social marketing to enhance uptake of condoms among sex workers, an approach that was found highly effective as part of the Avahan Initiative in India [18]. Social entrepreneurship models have slowly gained acceptance in a number of public health fields [19],[20], but are particularly important in sexual health where point-of-care tests are increasingly affordable and available. Expanding point-of-care HIV testing is a key priority within the WHO/UNAIDS Treatment 2.0 Global Strategy [21]. Technological advances in point-of-care testing for HIV and syphilis [22]-[25] facilitate decentralized services and provide unique opportunities for new social entrepreneurship models.

**Table 1:** **Core values of social entrepreneurship for sexual health**

Core value	Assessing core values
*Sexual health promotion*	Does the initiative primarily focus on promoting sexual health, defined as a state of physical, mental and social well-being in relation to sexuality?
*Multi-sectoral engagement*	Does the initiative engage key sectors needed for an effective multi-sectoral response (public health, business, marketing, technology, social change, academic, medical, law/regulatory)?
*Horizontal organization*	Does the initiative directly respond to community directives and empower key populations?
*Innovation*	Does the initiative authentically deliver additional value/benefit beyond what is currently available?
*Accountability*	Does the initiative have a clearly defined organizational structure, including an external advisory board that meets regularly?
*Balancing public health and entrepreneurial benefits*	Does the initiative have explicit benchmarks for public health and entrepreneurial success? How are these two core components balanced?

Translating the overarching SESH framework into actionable programs requires a detailed understanding of existing organizational and financial characteristics of HIV/syphilis service delivery in addition to evaluation of new pilot programs. Organizational characteristics are critical because they establish the human resources, technical capacity, and networks and alliances that ultimately have a large influence on effective sexual health programs [26]. In turn, financial characteristics demonstrate the ability for an organization to generate revenues and sustain service provision [27]. We examined organizational and financial characteristics of conventional HIV/syphilis testing for MSM and new pilot programs focused on revenue-generation for sustainability.

## Methods

### Ethics statement

This study was approved by the Institutional Review Boards of the Guangdong Provincial STD Control Center, the London School of Hygiene and Tropical Medicine, and the University of North Carolina at Chapel Hill. Involvement of investigators from Harvard University, Columbia University, and Sun Yat-sen University was arranged with respective IRBs. All participants provided verbal informed consent because the study was minimal risk and obtaining written signatures would be unusual in the Chinese context. This consent procedure was approved by all respective IRBs.

### Study population and setting

MSM comprise almost one third of China's new HIV cases [28]. HIV prevalence has risen sharply among MSM to a national average of 6.3% and exceeds 15% in some urban areas [28]. High levels of unprotected sex [28]-[31], partner concurrency [29],[32],[33] and ulcerative STDs such as syphilis [34] fuel the expansion of sexually transmitted HIV among MSM in China [35].

Free HIV and syphilis testing is provided in many government and community-based settings in China [36], but stigma, discrimination and fear may keep MSM from testing [37]. Despite national efforts that have increased HIV voluntary counseling and testing [38], 2011 surveillance estimates found that only 50.4% of MSM nationally had received an HIV test in the past year and knew their serostatus. MSM behaviors in China are highly stigmatized, decreasing disclosure of sexual orientation to family, friends, and health professionals [39]. The closeted identity of many MSM may compromise testing service delivery [40].

Our data collection focused on two cities in South China — Guangzhou and Hong Kong. The southern province of Guangdong is one of the six provinces in China with the highest reported number of HIV cases and has seen a particularly dramatic increase in syphilis since 2004 [41]. There are an estimated 200,000 MSM in the Pearl River Delta region of Guangdong, and prevalence of HIV and other STDs is higher among MSM in Guangdong Province (HIV: 7% [42], syphilis: 17% [43]) compared to national data.

Our study included MSM and policy stakeholders. MSM were defined as any biologically born male who reported ever having sex with other men. Policy stakeholders, referred to as stakeholders, were defined as those who have influence over HIV or syphilis policy and programs within the region. Both MSM and stakeholders were recruited in collaboration with local MSM CBOs, HIV organizations, hospitals, and public health clinics that serve the MSM population. Chinese individuals 16 years or older can legally provide consent and were eligible for recruitment. MSM were recruited in three ways - from government-sponsored health clinics, CBOs, and online through a popular MSM website. We used purposive sampling to recruit men across a variety of characteristics including age, socioeconomic group, sexual self-identity, marital status, and HIV/syphilis testing experience (first time, never tested, regular tester). Stakeholders were recruited from diverse fields including public health, business, technology, MSM CBOs, academic institutions, clinical medicine, law, and communications.

We used one-on-one interviews in order to reduce social desirability bias and better protect participant privacy and anonymity. Interviewers used a semi-structured interview guide but remained flexible for exploring new themes and accommodating the comfort level of each participant. Interviewers included an anthropology trainee, two public health researchers with formal training in anthropology, and two medical trainees who received qualitative research training. Interview guides were collaboratively developed and tested based on formative research among seven MSM and four stakeholders (data not included in the final analysis).

MSM interviews were conducted in private rooms at HIV testing centers or at a private location of the participant's choosing. The interview guide included the following core topics: sociodemographic information, HIV/syphilis testing experiences and preferences, stigma associated with HIV/syphilis and testing, facilitators and barriers to testing, social context of testing, and experiences with MSM-focused organizations and services. The stakeholder interview guide included questions about the stakeholder's background, experiences, challenges and advice regarding the following core topics: building local networks, developing and promoting MSM-specific services, HIV/syphilis testing, working with or at CBOs, funding and financing, local non-governmental organization capacity, and demographics (See Additional file 1: Interview guide). Data were analyzed using a Framework Analysis [44] developed by the UK National Centre for Social Research that incorporates both *a priori* themes as well as themes that emerge as part of the data collection process. Two coders independently coded each transcript using Atlas.ti 7.0 software guided by a structured codebook. The codebook was developed by the full research team to include a list of codes for all *a priori* and emergent themes, definitions of each code, and illustrative quotes to support each definition.

After all of the interviews were coded and discrepancies resolved, matrices were used to organize the themes by differential categories, particularly by type of HIV/syphilis testing organization, financial models, and new pilot testing programs. Patterns between themes were assessed to identify relationships across and within the differential categories. Coded quotes were chosen by group consensus to illustrate the variation and most common responses within each theme. The findings are presented below in three sections: organizing HIV/syphilis testing, financing HIV/syphilis testing, and pilot HIV/syphilis testing programs. Predetermined SESH core values (sexual health promotion, multi-sectoral engagement, horizontal organization, innovation, accountability, and balancing public health and entrepreneurial benefits ) provide benchmarks to evaluate pilot programs (Table 1) [45].

## Results

A total of 35 MSM and 49 stakeholders participated in the research study. Among MSM, 13 were between 16 and 25 years old, 20 were between 26 and 40 years old, and two were 40 years or older. Two MSM had less than high school education, eight completed high school only, and 25 had education beyond high school. Twelve MSM were originally from within Guangdong Province and 11 were from other provinces. Twenty-four MSM were currently working, seven were students, and three were not currently working. Twenty-seven MSM identified as Han and three reported other ethnicity. Twenty-eight MSM identified as gay, four as bisexual, and three as not sure or not reported. Seven MSM had never tested for HIV or syphilis, seven MSM had just undertaken their first test, and 21 MSM reported more than one test in the past.

Stakeholders included individuals from the following fields: 35 from a variety of CBOs, seven in medicine and public health, three in point-of-care testing technology, two from private business and enterprise, one academic professor, and one communications specialist. CBOs typically had two to eight permanent staff, several years of experience implementing point-of-care HIV/syphilis testing, and no formal advisory board. Among stakeholders, 37 were directly involved in creating HIV or syphilis testing programs or policies and 12 were indirectly involved in programs or policies.

### Organizational characteristics of HIV/syphilis test delivery models

There was substantial diversity in the organizations providing HIV/syphilis testing, including independent CBOs, independent clinics (e.g. hospitals, CDC-based testing), and hybrid CBO-clinic models (Table 2). CBO-clinic hybrid organizations are supported and organized by the CBO in collaboration with a local clinical service organization such as a city-level Center for Disease Control or hospital. Analyses identified advantages and disadvantages in terms of delivering HIV/syphilis tests through each of these three types of organizations.

**Table 2:** **Three organizational models (CBO, clinic, CBO-clinic hybrid) providing HIV/syphilis testing in South China**

Model	Description of organization	Advantages for delivering HIV/Syphilis testing	Disadvantages for delivering HIV/Syphilis testing
**Independent CBO**	CBO provides anonymous MSM testing, and has minimal formal relationship with clinic partners.	Good reputation among MSM , perceived safe environment , strong community	MSM may not trust medical services or tests compared to an independent clinic.
			MSM may see CBO as social meeting place, rather than testing center.
**Independent clinic**	Standard HIV/syphilis testing in a clinical setting (i.e. hospital/CDC center). Clinic may offer some social outreach activities and preventive services.	Trusted and standardized medical services, some MSM may feel services are anonymous because STD testing patients are mixed in with the general population	Poor communication and counseling, perceived discrimination, lack of confidentiality and privacy.
**CBO-clinic hybrid**	Shared decision-making, finances, staffing, space, and information between CBO and clinic.	Confidentiality and anonymity, high quality counseling and psychosocial support, perceived safe environment, medical services are trusted because standardized by government, trusted staff	Some communication issues between clinic providers and MSM.

CBO: community-based organization; MSM: men who have sex with men.

Independent CBO implementation of HIV/syphilis testing leveraged the client-centered environment of the CBO:

*I think especially in gay friendly organizations, they won't humiliate you or say anything, that makes you feel uncomfortable, so I don't feel any loss of face or anything like that… in organizations like this, I have never had that experience*. – *MSM-02, City B*

The CBO provided a safe environment sanctioned by the MSM community to provide point-of-care HIV/syphilis testing. However, some MSM were concerned about the quality of tests performed outside of government-sponsored clinics and may perceive independent CBOs as social meeting places rather than testing sites.

At the other end of the organizational spectrum, independent clinic-based HIV/syphilis testing provided technically competent, standardized services.

*Frankly speaking, I would go to some regular and authoritative organization like famous hospitals for that [testing]… Because it is more convincing and I would feel secure. There would be fewer mistakes. This is serious stuff so you should choose a professional organization. – MSM-15, City A*

Some MSM felt the process of getting tested at a clinic was more anonymous because they did not have to associate with a small, MSM-focused CBO.

*Some people will think that more people is better… in large hospitals, there are a lot of departments like gynecological, surgical, pediatric. Therefore he would think that with so many people mixed up, no one would notice him. – MSM-01, City A*

Yet the services provided at independent clinics were also identified as having poor communication and counseling (described by 3 men) and discriminating against MSM (described by 4 men). Several men (n = 3) described a lack of privacy and confidentiality in hospitals:

*They have done a poor job of keeping data confidential in the CDC and hospitals… there is a great risk of leaking names of special patients to the public, especially to the crowd queuing behind you. Some nurses may even shout to you “come in, Mr. XXX”… it is embarrassing for them to expose your name” – MSM-07, City A*

MSM also frequently perceived discrimination among doctors and nursing staff in hospitals:

*Doctors there [public hospital] weren't very friendly; they thought that since you are testing, something could be wrong with your personality…. Yes. Their attitudes were not very good. Because most of the people who go there for HIV testing are sex workers, drug dealers and drug addicts. - MSM-12, City A*

Respective organizational advantages of CBO and clinic-based testing services were both present in hybrid CBO-clinic testing sites. These organizations shared staff, space, financing, and strategic planning between local public health agencies and MSM CBOs. Hybrid organizational models combined the safe environment and client-centered atmosphere of the MSM CBO with the technical competence and standardized procedures of clinic-based services. Formally employed clinic staff were seconded to hybrid organizations and typically involved in drawing blood, performing tests, and collecting data. CBO staff at hybrid organizations focused on counseling services, result notification, linkage to treatment, and referring infected individuals to care. Collaboration between CBO and CDC-supported testing facilities made services more trusted compared with independent CBOs:

*Although this organization [hybrid CBO-clinic] is a gay organization, it's based on the medical stuff… it is cooperating with this CDC station. I just don't trust any organization that is only run by a gay group - MSM-02, City A*

Enhanced counseling services available at hybrid organizations facilitated the formation of deeper connections between MSM and CBO staff:

*I feel that this community is very good… And the staff here are very concerned about you. They would ask me something about my life… I think of them as my close friends and tell them lots of things without reservation - MSM-24, City A*

This client-facing environment linked with the technical competence of the clinical authority created a strong foundation for HIV/syphilis service delivery. From an MSM perspective, hybrid testing sites were responding to community needs and fulfilled an obligation to the community. From a clinical perspective, hybrid testing sites allowed increased case detection among a high-risk group who had a disproportionate disease burden. These hybrid organizations were able to successfully leverage the close connections between the CBO and the local clinics to provide useful services.

### Financial characteristics of HIV/syphilis test delivery models

Financial plans for operating HIV/syphilis testing services were homogenous across organizational models and dependent on unstable sources of funding. HIV/syphilis testing offered within clinic-based settings included fee-for-service hospital-based testing and government-subsidized HIV/syphilis testing services while testing at MSM CBOs was government-funded (local or provincial public health departments) either through public health clinics directly providing tests or through subcontracts to CBOs. Although such testing services were generally provided to clients without charge through government subsidy, there were no mechanisms to ensure sustainability of CBO testing programs and CBOs generally struggled to find the additional funds needed to cover their operating expenses (e.g. testing space rental, phone lines, outreach, staff stipends/salaries). Ad hoc support to CBOs for HIV/syphilis testing from global biotechnology companies or foundations created a culture of CBO dependency:

*And now we [an MSM CBO] suffer. In the new round of AIDS Trust Funding, they decided not to fund any of our STD testing services because they think it is only a gimmick… but in the old days they funded all of our STD testing services, but now they only cover HIV tests. - Stakeholder-18, CBO*

CBO leaders also stated that current grant funding sources made it difficult for them to maintain their own objectives and organizational identity:

*Their [CBO] priorities tend to reflect donor priorities, so that they can secure funding…What tends to happen is that they just adopt whatever the objectives of a project are in order to satisfy what they think the donor wants to see. - Stakeholder-4, CBO*

All MSM CBOs mentioned that cutbacks in external support for STD testing services were forcing organizations to reduce or alter testing services.

Interviews with stakeholders revealed that the critical function of the MSM CBO to authentically represent the community has been partially under-cut by CBOs' financial dependence on other organizations. Restrictions on hiring staff or paying for other administrative fees and operating costs (e.g. space rental fees, phone lines, etc.) as part of many external funding schemes compromised the ability of CBOs to maintain or scale up testing programs. Exorbitant fees associated with the official registration process for non-governmental organizations precluded all mainland MSM CBOs from being officially registered as non-profit organizations, limiting their fundraising capabilities:

*CBOs can't raise money or receive donations. All of their funds are from grants unless they are registered and it is very difficult to register. Besides, you need to register at a specific level. For example, you'd pay 30,000 yuan to register for one distinct, 60,000 yuan for one city, 300,000 yuan for one province. If we register for our district, we can't work in another district. But people come to our organization from all over the province! - Stakeholder-9, CBO*

### Pilot programs to generate revenue for sustainability

None of the independent CBOs or independent clinics that we studied had organized revenue-generating testing programs. Four hybrid CBO-clinic testing sites included in our analysis piloted revenue-generating HIV/syphilis testing programs intended for MSM. We interviewed eight MSM and seven stakeholders who had experience with these programs. In all four cases, the revenue-generating pilot testing programs had been undertaken out of necessity caused by increasing demand for HIV/syphilis services alongside decreasing external financial support. Revenues generated from pilot programs resulted from the sale of individual products (e.g., condoms, point-of-care HIV/syphilis tests) or services (e.g., specialized clinical services) (Table 3). Pilot programs either directly linked revenue generation and public health through selling health-related products (Table 3A) or services (Table 3B) or had independent revenue generation in order to support subsequent public health components (Table 3C and D).

**Table 3:** **HIV/syphilis testing pilots focused on revenue generation for sustainability**

	Product-based enterprise (selling a product)	Service-based enterprise (selling a service)
**Direct social benefit**	**A**	**B**
	**Direct product-based enterprise**	**Direct service-based enterprise**
	Revenue-generating social product has direct social value.	Revenue-generating key population-friendly health services delivered in a comfortable setting
	Examples:	Examples:
	Sales of condoms , sales of point-of-care STD tests	Private clinic tailored to FSW as a well-women's clinic
**Indirect social benefit**	**C**	**D**
	**Indirect product-based enterprise**	**Indirect service-based enterprise**
	Portion of revenue generated from selling a product unrelated to social cause invested in a social cause.	Portion of revenue generated from selling a service unrelated to social cause invested in a social cause.
	Examples:	Examples:
	Clothes, underwear, swimming suit shop, book shop	Online advertisements, partnerships with businesses

STD: sexually transmitted disease; FSW: female sex worker.

The longest running pilot program focused on revenue generation through sales of point-of-care HIV and syphilis tests at a CBO. This new service provided value by offering rapid oral HIV tests which are not available at conventional testing sites, facilitating online appointment booking, and ensuring high-quality counseling services. Individuals paid 16 USD per HIV test, slightly greater than the bulk-cost of the test. During the first three months of operation, this program provided testing services to 67 individuals and identified three individuals with newly diagnosed HIV infection. Men who received testing through this pilot program felt that they were receiving high-quality services. Seven MSM indicated that paying for STD tests should be considered normal in a health care setting that is primarily fee-for-service:

*There is nothing I can do about it if fees need to be charged… After all this is about my own health. -MSM-17, City A*

Two MSM referred to the inevitability of fee-for-service testing given the current economic climate. Three MSM compared paying for STD tests to paying for other routine blood tests.

While the pilot revenue-generating programs we identified demonstrated the potential for sustainability, none of the programs adhered to all of the SESH core values (Table 1). Among the four pilot sites assessed, core values related to sexual health promotion, multi-sectoral engagement, and horizontal organization were upheld. From an innovation perspective, although all four pilot programs employed creative revenue-generation components (sales of condoms, sales of advertisements), only the oral point-of-care HIV test pilot offered a truly innovative sexual health service beyond what is currently available to MSM in the local area. In addition to offering oral HIV tests, other innovations to existing testing services discussed by MSM and stakeholders included, out-of-hours testing, enhanced counseling and accompaniment to appointments, and self-testing. From the perspective of accountability, none of the pilot programs had a clearly defined organizational structure with regular input from an external advisory board. Finally, none of the pilots had explicit goals or performance benchmarks in order to evaluate and balance their public health and entrepreneurial goals.

There are several risks associated with using social entrepreneurship to expand HIV/syphilis testing. Several MSM and stakeholders noted that a social entrepreneurship project could create confusion among testers unless clearly re-branded from existing CBO and government services. Some MSM and stakeholders were also concerned that paying for testing commercializes a service that has traditionally been provided through the public sector.

In addition, new pay-for-service models could inadvertently thwart testing, especially among subsets of MSM with limited income (e.g., students, migrant workers, unemployed). Some men in our sample noted that paying for testing would either discourage testing (n = 2) or decrease the frequency of testing (n = 6). None of the pilot programs underway had formally evaluated these potential unintended consequences of using revenue-generating models.

## Discussion

The acute effects of global economic uncertainty alongside chronic under-funding of key population sexual health services have fundamentally changed HIV/syphilis testing in many regions. For example, the United States Centers for Disease Control and Prevention anticipated supporting 424,000 fewer HIV tests in 2013 due to budget cuts [46]. Local services must make do with fewer resources or substantially reduce the extent of routine screening services provided. In this context, social entrepreneurship may support more sustainable sexual health service provision. Most social entrepreneurship research has focused on poverty alleviation [47],[48] and small business development [15], with far less research focused on improving health. Newly developed point-of-care tests for HIV, syphilis, and other STDs [24] alongside the increasing capacity of MSM CBOs to deliver these tests create opportunities to pilot sustainable social entrepreneurship models. These models for providing testing services could be relevant to many low and middle-income settings where public sector services incompletely reach key populations.

Our data demonstrates a wide range of organizations providing HIV/syphilis testing services. Hybrid CBO-clinic testing sites effectively integrate the client-facing inclusive environment of the MSM CBO with the technical competence and facilities of a public health clinic. Although point-of-care HIV and syphilis tests have only been on the market in China for a few years, they have rapidly been incorporated into hybrid CBO-clinic testing sites [49]. In one South China city, hybrid CBO-clinic HIV testing accounts for approximately 40% of all MSM tested and 60% of all newly diagnosed HIV cases [50]. While similar hybrid CBO-clinic service models are sometimes found in high income nations [51],[52], these types of organizations are uncommon in low and middle income nations that have substantial MSM HIV epidemics.

Hybrid CBO-clinic MSM organizations have responded to financial shortfalls by piloting revenue-generating projects. SESH pilots ranged from selling condoms to more elaborate key population-friendly clinical services or point-of-care tests. Sales of point-of-care HIV and syphilis tests are particularly worthy of further consideration given that they could be implemented at decentralized locations with minimal staff training [53], have high sensitivity and specificity [54], and provide an entry-point for subsequent sexual health services [55]. Transitioning revenue-generating operations into full-fledged social enterprises will require capacity building focused on innovation, accountability, and evaluation (Table 1). Identifying and selling point-of-care tests to the massive population of untested high-risk individuals will require a marked departure from conventional services. Given the high level of government resources focused on HIV testing among MSM in China, hybrid CBO-clinic organizations may be able to leverage public sector support for models that are locally trusted and highly utilized by MSM.

Our data identify modifiable risks associated with using social entrepreneurship to expand HIV/syphilis testing. Foremost among these potential risks is misunderstanding a new social entrepreneurship project as a purely profit-motivated enterprise. This confusion has been noted in other social entrepreneurship projects [56],[57] and could be alleviated through including communications expertise in project design, promotion, and implementation. In order to mitigate this risk, social entrepreneurship programs will need to define the relationship between their public health and entrepreneurial priorities and ensure that these priorities are reflected during implementation.

Our study has several important limitations that could be kept in mind when inferring about other local contexts. First, this research was undertaken in China where there are limits on the extent to which organizations can be formally registered as an NGO and few domestic foundations exist to assist NGO development. However, many low and middle income nations have nascent NGO sectors [58] and few foundations [59], increasing the need for new models that promote social change and opening the door for social entrepreneurship. SESH programs will need to assess the local landscape of services in order to be relevant and innovative. Second, our research focused on MSM and did not collect data from other key populations. At the same time, microenterprise research from women's groups and sex worker collectives in other parts of the world [60] increases the likelihood that a social entrepreneurship framework could be productive among other key populations. Third, our results are formative and do not comprehensively evaluate all aspects of the new social entrepreneurship models. More refined metrics for investigating social enterprise development are needed in order to weigh their respective advantages.

## Conclusion

Sustainable HIV/syphilis services are essential for MSM and other key populations, but current service delivery financial models are challenged by budget cuts and uncertainty. Social entrepreneurship initiatives can help reorganize sexual health services in order to make them more sustainable and community-driven. Our research shows that social entrepreneurship models will not be panaceas, but could be a useful adjunct to promote HIV testing. Further implementation research is needed to guide the development of social entrepreneurship pilot programs focused on improving sexual health.

## Additional file

## Electronic supplementary material

Additional file 1: Stakeholder Semi-Structured Interview Guide (with sexual health experience). (DOCX 19 KB)
