# Supplementary material for: Organizational characteristics of HIV/syphilis testing services for men who have sex with men in South China: a social entrepreneurship analysis and implications for creating sustainable service models
Source: BMC Infect Dis. 2014 Nov 25;14:601. doi: 10.1186/s12879-014-0601-5 (PMC4247875; doi:10.1186/s12879-014-0601-5)
Supplement: Supplementary file 1 — Additional file 1: Stakeholder Semi-Structured Interview Guide (with sexual health experience). (DOCX 19 KB) [file 12879_2014_601_MOESM1_ESM.docx]

**Stakeholder Semi-Structured Interview Guide (with sexual health experience)**

Informed consent

Context of this research

Interview topics (below)

**Background questions for each interviewee (warm-up)**

1. Can you talk a little bit about your role at this organization/company/lab/etc?
   1. Probes: How long have you worked here?
2. What is your past experience in sexual health care delivery?
3. What is the organizational structure of the company/lab?
   1. In your opinion, does the organization adjust well to change? Can you give an example?

**Building local networks**

1. Describe the working relationship that your organization has with private companies, public sector, or other organizations.
   1. Are there other organizations that your organization would like to work with in the future?
   2. What kinds of difficulties have you experienced in trying to partner with certain organizations?
   3. Are there organizations that you avoid working with and why?
2. How can local partnerships be expanded to promote MSM HIV/syphilis control?
3. What are the barriers to broader partnerships focused on MSM issues? Why do you think these barriers exist?

**MSM HIV/syphilis experience**

1. Describe some of the current HIV/syphilis control efforts that you and/or your organization are most involved with or knowledgeable about?
   1. Rate of testing
   2. Outreach methods
   3. Describe your idea of a successful sexual health campaign that your organization or another organization has been involved in.
2. What are your major concerns regarding the current HIV/syphilis control efforts of your organization?
   1. How do you think your organization can improve the rates of HIV/syphilis testing?
3. Which MSM are likely to access current services and which types of men do not?
   1. What factors contribute to this?
   2. How has it changed since you have worked here?
4. What does your organization provide for MSM HIV/syphilis testing, linkage, and retention?
   1. How has this changed over time?
5. Do you know of anyone who has been personally affected by the need for increased sexual health care and testing?

**CBO Roles (first define community based organization)**

1. What are appropriate roles for MSM CBOs in organizing HIV/syphilis response?
2. How can the following types of risk and challenges associated with SESH be decreased?
   1. How can we address potential regulatory problems around obtaining point-of-care diagnostic tests and their use in non-clinical settings?
   2. How can we maintain the reputation and identity of individual organizations under the SESH initiative?
   3. How can we maintain a clear organizational structure between the various sexual health delivery organizations?
   4. How can we meet unfulfilled demand and ensure complete follow-up while maintaining optimal patient/customer experience?
   5. How can we avoid confusion related to the varying financial incentives of different sectors?
3. What do you foresee as additional potential challenges in the SESH initiative?

**Facilitators and barriers to HIV/syphilis testing**

1. What would make it easier for you to offer syphilis testing routinely for all STI patients?
2. What would make it easier for you to offer HIV testing routinely for all STI patients?
3. What would make it difficult for you to offer syphilis testing routinely for all STI patients?
4. What would make it difficult for you to offer HIV testing routinely for all STI patients?
5. Discuss the following reasons for (not?) offering syphilis/HIV testing:
   1. Guidelines
   2. Disease prevalence
   3. Cost
   4. Space
   5. Too busy to have time to test all patients
   6. I can’t ensure good follow-up for patients
   7. I don’t have enough training (discuss deficient training area)
   8. HIV stigma

**“Face” and STI Testing**

1. How will partnering on a project on HIV/syphilis testing affect your role in your organization or social group?
   1. Share any relevant experiences
2. If your friends and family knew that you diagnosed an STI patient with HIV, would that affect their impression of you as a doctor?

**Financing**

1. Who pays for syphilis and HIV testing in your city?
2. What are the major revenue sources for your organization, including public and private sources?
3. How sustainable are these revenue sources?

**Wrap Up**

1. Who else should we be talking to?
2. What issues (in addition to the one’s we’ve identified) might be important in the context of our research?
